# Eight-Year Follow-Up Using a Fresh Osteochondral Allograft for a Femoral Head Chondroblastoma in a 17-Year-Old Patient

**DOI:** 10.1155/2019/9262190

**Published:** 2019-09-09

**Authors:** Louis-Charles Moreau, Philippe Beauchamp-Chalifour, Etienne L. Belzile, Norbert Dion

**Affiliations:** ^1^Faculty of Medicine, Department of Surgery, Division of Orthopaedic Surgery, QC, Quebec, Canada; ^2^Department of Surgery, CHU de Québec-Université Laval, Hôtel-Dieu de Québec, 11, Côte du Palais, Québec, Quebec, Canada G1R 2J6; ^3^Centre de Recherche FRQS du CHU de Québec-Hôpital Enfant-Jésus, 1401, 18ème Rue, Québec, Quebec, Canada G1J 1Z4

## Abstract

Chondroblastoma is a rare benign tumor that affects the epiphysis of long bones in adolescents. Chondroblastoma located in the femoral head is associated with a higher recurrence rate and carries the additional risks of head collapse and degenerative hip disease. Aggressive curettage followed by bone grafting is the current mainstay of treatment. To our knowledge, the long-term postoperative outcome of this technique remains unknown due to the short follow-up of previous case reports. We present the case of a 17-year-old male who underwent fresh osteochondral allograft following curettage of a femoral head chondroblastoma, using a Ganz surgical hip dislocation. He made an uneventful recovery without tumor recurrence. The patient was followed up to 8 years postoperatively. However, there were clinical and radiographic degenerative changes at 6 years of follow-up.

## 1. Introduction

Chondroblastoma, also referred as Codman tumor, is a rare entity accounting for 1-2% of all bone tumors [1–3]. It usually presents as a painful epiphyseal lesion in the second decade of life and can be locally aggressive depending on its localization [3]. Male patients are affected twice as frequently as females [4]. The humerus, femur, and tibia are affected in 70% of cases [5]. Exceptional malignant transformation of these lesions has been reported [6]. Radiographically, chondroblastoma is described as an epiphyseal lytic lesion with a thin sclerotic rim [5]. Internal calcifications have been reported in 40-60% of cases [5].

Chondroblastoma located in the femoral head is associated with a higher recurrence rate and carries the additional risks of head collapse and degenerative hip disease [3, 7].

The goal of treatment is to remove the lesion and preserve the articular surface [3]. Many techniques have been described to address chondroblastoma of the femoral head, but aggressive curettage followed by bone grafting is the current mainstay of treatment [3, 8, 9].

Femoral head chondroblastoma is very rare, and due to the small number of reports, it is hard to draw conclusions about clinical outcome of this disease [10]. The long-term postoperative evolution of patients treated with bone grafting is unclear.

We present the case of a 17-year-old male who underwent fresh osteochondral allograft following curettage of a femoral head chondroblastoma, through a Ganz surgical hip dislocation. The patient was followed up to 8 years postoperatively. He made an uneventful recovery without tumor recurrence. However, he showed signs of hip osteoarthritis at 6 years of follow-up.

## 2. Case Report

A previously healthy 17-year-old man presented to our institution with a 1-year history of left hip pain and stiffness. There was no history of trauma or previous hip pathology. His symptoms had an insidious onset, exacerbated by physical activity. Pain was chronic and stabbing and radiated to the ipsilateral knee and ankle. The patient had intermittent limping. He had lost 10 pounds in the last year but denied any other constitutional symptoms. Three months of conservative management with nonsteroidal anti-inflammatory drugs (NSAIDs) and regular physical therapy failed to improve his condition.

Examination revealed slight left lower limb atrophy with a Trendelenburg gait. The affected hip range of motion was decreased, with 60° of hip flexion, 20° of internal rotation, 25° of external rotation, and 60° of abduction. Hip movements were uncomfortable. A leg roll test was positive. Neurovascular examination was normal.

**Figure 1:**
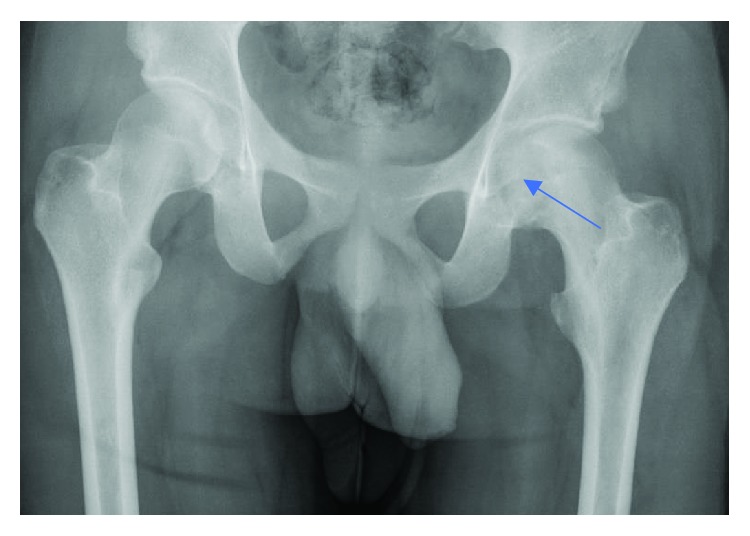
Preoperative AP pelvis X-ray showing femoral head osteolytic lesion (blue arrow).

**Figure 2:**
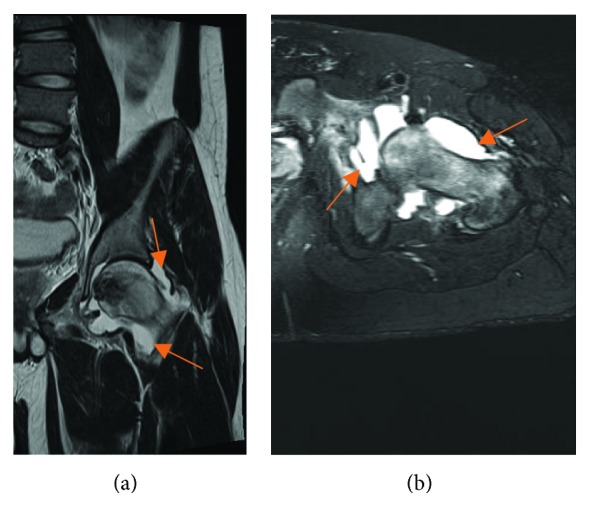
T2 MRI views of the left hip: coronal view (a) and axial view (b) of the hip with joint effusion (orange arrows).

**Figure 3:**
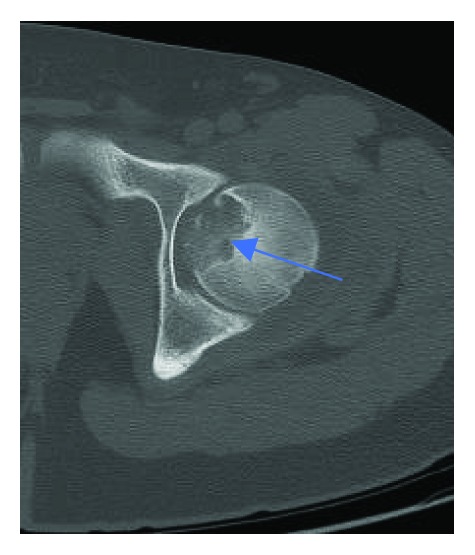
CT-Scan axial view illustrating a 3.5 × 3.2 × 2 cm lesion with sclerotic borders and intralesional calcifications (blue arrow).

Radiographs showed an epiphyseal lytic lesion of the proximal femur with the involvement of the fovea (Figure 1). Magnetic resonance imaging (MRI) showed a 3.4 × 2.6 × 3.2 cm (AP × T × C − C) femoral head epiphyseal lesion, with intra-articular extension. An associated joint effusion was noted, as well as bone marrow edema (Figure 2). A computerized tomography scan (CT-Scan) showed a well-defined lesion of 3.5 × 3.2 × 2 cm with sclerotic borders and intralesional calcifications suggestive of a chondroid-like matrix (Figure 3).

**Figure 4:**
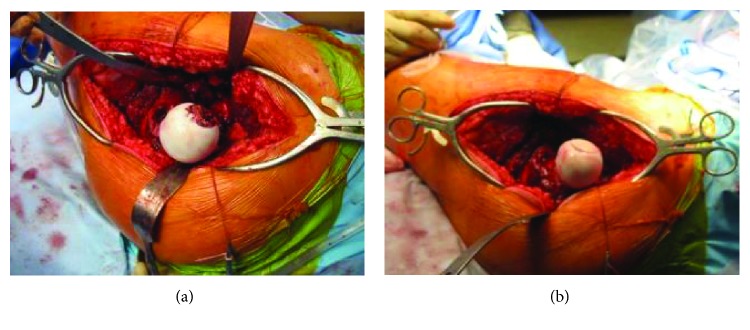
(a) Surgical view of the femoral head lesion following anterior hip dislocation. (b) Surgical view of the femoral head showing congruent graft surface with the radius of the host femoral head.

**Figure 5:**
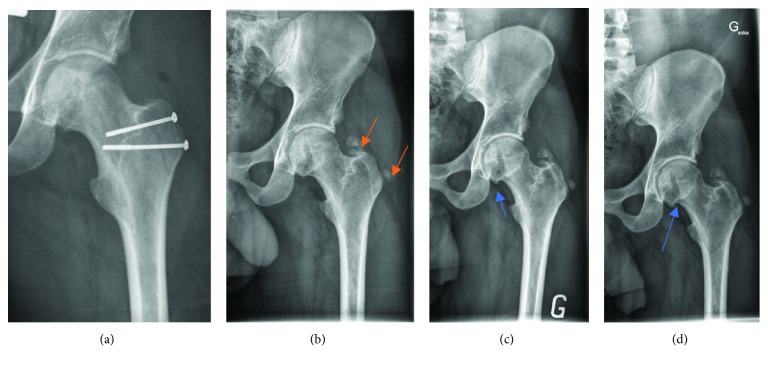
(a) Immediate postoperative AP pelvis radiography showing the two 4.0 mm cannulated screws stabilizing the greater trochanter osteotomy. (b) Two-year postoperative AP X-ray showing good graft position without head collapse or degenerative changes, as well as heterotopic ossification at the tip and lateral to the greater trochanter (orange arrows). (c) Six-year postoperative AP X-ray showing femoral head inferior osteophyte with narrowing of the joint space (blue arrow). (d) Seven-year postoperative AP X-ray showing discrete progression of femoral head inferior osteophyte (blue arrow).

The patient was scheduled for a Ganz surgical hip dislocation [11]. The hip was approached through a direct lateral incision. A greater trochanter chevron osteotomy was performed, followed by anterior capsulotomy. The hip was carefully dislocated anteriorly (Figure 4(a)). The round ligament was sectioned at its base upon exposure due to tumor invasion. An open biopsy was carried out. Frozen sections were highly suggestive of a benign cartilaginous lesion, compatible with chondroblastoma. There were no macroscopic damages seen intraoperatively on the articular cartilage. Aggressive curettage was then performed. We used the mosaïcplasty instrumentation, specifically a 30 mm trephine, to create a geometrical defect in the patient's femoral head, following the intralesional curettage of the tumor. A fresh osteochondral allograft ordered from the Joint Restoration Foundation (Centennial, CO, USA) was press-fitted into the resulting 30 mm wide cylindrical femoral head defect. The donor graft surface curvature was congruent with the radius of the host femoral head (Figure 4(b)). A calibrated spherometer was then utilized to make sure the graft was appropriately sunk in the defect to avoid impingement on the labrum. The hip was relocated into the acetabulum, and the greater trochanter osteotomy was stabilized with two 4 mm cannulated screws. The hip was mobilized intraoperatively and was found to be stable. Postoperative radiographs showed proper graft positioning and restoration of joint line congruity (Figure 5(a)).

**Figure 6:**
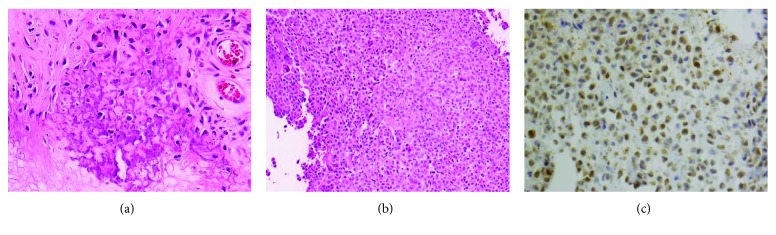
Histopathological sections of the lesion: (a) intracellular calcification, (b) mononuclear cells with a characteristic pale cytoplasm, and (c) positive S100 immunolabeling within chondroblast cells.

Toe touch partial weight bearing was allowed for eight weeks. Progressive transition to full weight bearing was allowed over the next 4 weeks. The two cannulated screws were surgically removed at 6 months. The pathologist confirmed the diagnosis of chondroblastoma on histopathological examination (see Figure 6).

**Figure 7:**
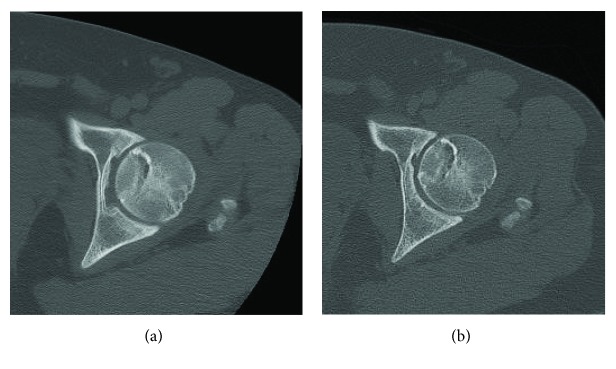
Axial CT-Scan of the left hip: (a) 2 years postoperatively and (b) 6 years postoperatively.

The follow-up at 3 months was uneventful. At 1 year postoperatively, the patient had gotten back to his normal daily activities, including jogging. At 2 years of follow-up, the patient complained of 5 episodes of hip locking. The hip was stable upon examination. He had up to 70 degrees of flexion. There was no limping. Radiographs and a CT-Scan confirmed proper graft incorporation (see Figure 7(a)). There was no tumor recurrence, no head collapse, and no degenerative changes (Figure 5(b)).

**Figure 8:**
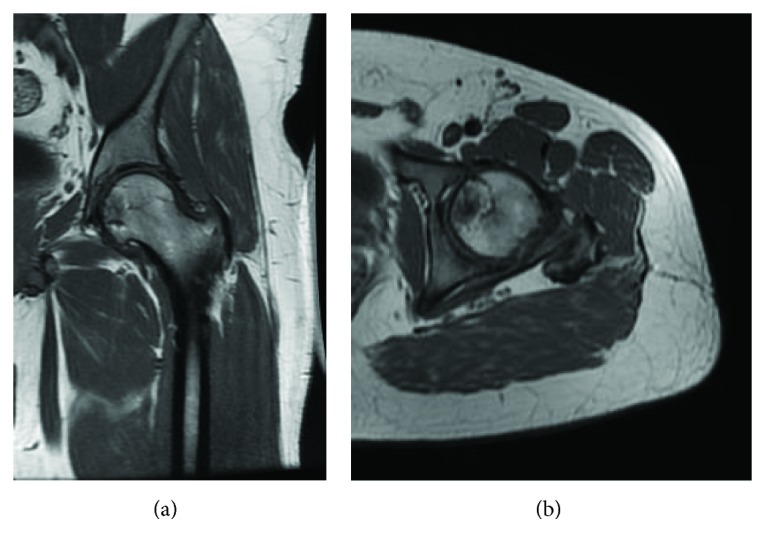
Postoperative MRI of the left hip at 6 years: (a) T1 AP view and (b) T1 axial view of a well-integrated osteochondral graft.

The patient stopped coming to his regular follow-up. He came back to our clinic at 6 years, complaining left inguinal pain while jogging (he tried to run 21 km without success). There was no limping upon examination. He could reach 90 degrees of flexion and had 5 degrees of external rotation (20 degrees on the contralateral side). On X-rays, there were minor degenerative changes with inferior femoral head osteophytes and joint space narrowing medially (Figure 5(c)). The osteoarticular allograft still looked intact on the CT-Scan images (Figure 7(b)). We advised him to stop running. At the 7th year visit, the patient complained of the same progressing left inguinal hip pain exacerbated by physical activity. Hip flexion was still at 90 degrees. There was minor progression of previously described degenerative changes (Figure 5(d)). Left hip MRI and scintigraphy were obtained to rule out infection, head collapse, or graft failure. There were no signs of local recurrence in the femoral head (Figure 8). We concluded his symptoms were caused by early hip osteoarthritis and secondary deconditioning. He was referred to physiotherapy. At 8 years, he still had pain, which responded well to a cortisone intra-articular injection.

## 3. Discussion

In skeletally mature patients, aggressive curettage of lesions in the proximal femoral epiphysis can be performed through a window in the femoral neck, or directly through the articular cartilage, with a trapdoor procedure [12]. More recently [13], Xu et al. have described a modified trapdoor approach, using the fovea as a window to address the lesion and the ligamentum teres to close the bony defect following curettage. In our case, we elected to use a surgical dislocation to have better access to the intra-articular extension of the tumor, compared with a cortical window in the femoral neck. We chose to use a fresh osteoarticular allograft, because we were concerned with the risk of impingement between the edges of the surgical defect and the labrum. It seemed like the most reasonable choice to achieve our goals of treatment, which were performing an adequate intralesional resection, as well as preserving joint surface and maximizing hip function in a 17-year-old patient. The restoration of the articular joint line following oncologic resection is of concern with large femoral head lesions due to the associated risks of head collapse and early osteoarthritis, especially in young patients [9, 14–16]. Fresh osteochondral allografts have been previously used successfully to fill articular surface deficits in the knees, ankles, shoulders, and hip joints [9, 14–16]. Hyaline cartilage from fresh grafts is avascular and aneural and receives its nutrition from synovial fluid, thus making it a good tissue to transplant [17]. Disadvantages of fresh grafts include risks of infection, diminished cellularity, and potential immunologic reaction [17–20]. Also, recurrence rates as high as 20% have been reported with chondroblastoma, thus making it imperative to do an aggressive curettage [3]. Frozen grafts would have been another graft option, but this method eliminates 95% of viable chondrocyte compared to fresh grafts [17–20]. We wanted to maximize the longevity of the femoral head by using the best graft available. A fibular vascularized graft has been used in a 36-year-old woman for a larger femoral head resection with excellent functional outcomes at 9 years of follow-up [21]. Radiofrequency ablation could represent a less-invasive treatment modality but has only been used in small lesions [22, 23]. Joint arthroplasty is another option for treatment but in a 17-year-old would have been limited in terms of function, and we would have exposed him to revision surgery in the future [8, 9, 24]. Curettage alone or the removal of the lesion without bone graft is another solution, but this would have exposed the patient to the risk of impingement between the edges of the surgical defect and the labrum, with earlier hip osteoarthritis because of the subsequent articular surface destruction [8, 9, 24]. Reported techniques and outcomes for femoral head chondroblastoma are summarized in Table 1.

Proximal femoral lesions are particularly at risk of recurrence, perhaps due to technical difficulties with proper access and complete excision [3]. While various hip approaches can be used, the surgical dislocation technique described by Ganz offers appropriate joint visualization while minimizing the risk of avascular necrosis (AVN). This technique spares the medial femoral circumflex artery [11]. In a series of 213 hips surgically dislocated with this technique, they did not encounter AVN (follow-ups ranging from two to seven years postoperatively). A prospective trial describing 28 patients undergoing surgery for a Pipkin fracture through Ganz surgical dislocation did not show any AVN at 36 months of follow-up [25]. A 37% rate of heterotopic ossification at one year has been reported with this technique, as seen in our case at 2 years postoperatively (Figure 5(b)).

We report a femoral head chondroblastoma in a 17-year-old man that was successfully treated with a fresh osteochondral allograft, following an aggressive curettage through a Ganz surgical dislocation. To our knowledge, our case represents the longest follow-up available in the literature for this surgical technique. At 8 years of follow-up, we observe radiological incorporation of the graft with joint line congruity, as well as no signs of head collapse or avascular necrosis. There is no local recurrence despite a high-risk primary site and initial intra-articular extension. However, there are clinical and radiographic signs of early osteoarthritis at 6 years postoperatively. The patient admitted doing repeated shock activity (long-distance running), which we recommended to be stopped. The patient still has good hip function. With early hip osteoarthritis at 23 years of age, this patient may need a joint arthroplasty in the future. A recent study by Farfalli et al. [7] reported a 38% incidence of secondary arthritis in their 53-patient cohort treated for chondroblastoma, more specifically in 5 of the 8 proximal femoral lesions. Four of these patients were eventually treated with a total hip replacement, at an average 120 months following the index procedure. Joint cartilage 5-year survival was calculated at 90% for all lesions, but only at 44% for lesions in the femoral head.

We conclude that fresh osteochondral allografts through a Ganz surgical dislocation can be a good solution for a femoral head lesion with a favorable outcome at 8 years. However, low-impact physical activity and physiotherapy should be recommended to patients undergoing these types of intervention in order to minimize early osteoarthritis risks and improve hip function. Recent literatures suggest that a joint cartilage has a worse prognosis when the lesion is located in the femoral head.

## Figures and Tables

**Table 1:** Reported techniques and outcomes for femoral head chondroblastoma^∗^.

Technique	Outcome
*Curettage alone*	
(i) Trapdoor procedure [12] (ii) Modified trapdoor procedure [13] (iii) Surgical dislocation [11]	(i) Removal of the lesion (ii) Risk of impingement between the edges of the surgical defect and the labrum (iii) Articular surface destruction (iv) Earlier hip osteoarthritis [8, 9, 24]
*Allograft following curettage*	
(i) Fresh osteochondral allografts [9, 14–16] (ii) Frozen grafts allografts [17–20] (iii) Fibular vascularized allograft [21]	(i) Restoration of the articular surface (ii) Higher risk of infection and potential immunologic reaction [17–20] (iii) Limited data available using those techniques
*Radiofrequency ablation* [22, 23]	Efficiency only described in small lesions
*Arthroplasty* [8, 9, 24]	Total joint replacement

∗Recurrence rate as high as 20%, this making it imperative to do an aggressive curettage [3].
